# Supplementary material for: A k-mismatch string matching for generalized edit distance using diagonal skipping method
Source: PLoS One. 2021 May 4;16(5):e0251047. doi: 10.1371/journal.pone.0251047 (PMC8096107; doi:10.1371/journal.pone.0251047)
Supplement: Attachment — Submitted filename: PONE-D-20-11239-rebuttal(201130).pdf [file pone.0251047.s001.pdf]

**Dear Editor**

Thank you for offering the opportunity to revise the paper PONE-D-20-11239. The paper has been revised with careful study in aspects of paper format, language, terminology, conveyed meaning, and grammar. In addition, the revised paper addressed academic editor and all reviewers' comments.

============================================================

**Journal requirements**:

1. When submitting your revision, we need you to address these additional requirements. Please ensure that your manuscript meets PLOS ONE's style requirements, including those for file naming. The PLOS ONE style templates can be found at main_body.pdf and title_authors_affiliations.pdf"

   ***Answer***: According to the journal requirements mentioned above, our manuscript has been reviewed and revised considering whether this revision meets the PLOS ONE's style or not. In addition, new PLOS ONE latex template and its format has been applied to this revision.

2. We suggest you thoroughly copyedit your manuscript for language usage, spelling, and grammar. If you do not know anyone who can help you do this, you may wish to consider employing a professional scientific editing service.

   ***Answer***: In agreement with the journal requirements and reviewer #1 concerns, this manuscript has been proofread by a scientific editing service. Besides, grammatical errors and typos have been revised in this version. I have attached the correction statement of this paper as:

KAIST
Language Center

(305-701) 대전광역시 유성구 대학로 291, Daehak-ro, Yuseong-gu, Daejeon 305-701
language@kaist.ac.kr, http://lang.kaist.ac.kr, Tel. 042-350-8731~2, Fax. 042-350-8730

# Proofreading Confirmation

| | |
|---|---|
| **Registration Number** | CR-20-2507 |
| **Date of Registered** | 2020. 10. 22 |
| **Title** | A k-Mismatch String Matching for Generalized Edit Distance using Diagonal Skipping Method |
| **Proofread Language** | English |
| **Author** | |
| **Proofreader** | Jim Collins |

**This paper was proofread by the KAIST Language Center**

Nov. 02, 2020

## KAIST Language Center

Korea Advanced Institute of Science and Technology (KAIST)

==============================================================

**Reviewer 1**:

Thank you for offering the opportunity to revise the paper PONE-D-20-11239. The paper has been revised with careful study in aspects of paper format, language, terminology, conveyed meaning, and grammar. In addition, the revised paper has addressed your concerns.

**Comments and Response:**

1.-While most of the paper ist written in understandable English, it is clear through the whole paper that the writer is not a native English speaker. I recommend having the paper checked by a native speaker or someone with experience in writing English manuscripts.

***Answer***: In agreement with your concerns, this manuscript has been proofread by a scientific editing service. Besides, grammatical errors and typos have been revised in this version.

KAIST

Language Center

(305-701) 대전광역시 유성구 대학로 291, Daehak-ro, Yuseong-gu, Daejeon 305-701

language@kaist.ac.kr, http://lang.kaist.ac.kr, Tel. 042-350-8731~2, Fax. 042-350-8730

## Proofreading Confirmation

| | |
|---|---|
| **Registration Number** | CR-20-2507 |
| **Date of Registered** | 2020. 10. 22 |
| **Title** | A k-Mismatch String Matching for Generalized Edit Distance using Diagonal Skipping Method |
| **Proofread Language** | English |
| **Author** | |
| **Proofreader** | Jim Collins |

**This paper was proofread by the KAIST Language Center**

Nov. 02, 2020

**KAIST Language Center**

Korea Advanced Institute of Science and Technology (KAIST)

2. The description of the algorithm is hard to read, mainly because some terms are used before they are explained. Especially the term "arrow" is not defined explicitly. Also, the pseudocode states an implementation step, but the reader has to search for a more detailed description of this initialization.

***Answer***: Thanks for your comments. For more readability, we have renewed the explanation of the arrow and its usage of diagonal traversal in lines 229~240 as:

> In Fig 4 (b), diagonal traversals are illustrated, where the arrow indicates the order of steps calculated in each traversal. Fig 4 (b) describes that the upper right step is calculated before the lower left step in a diagonal traversal. It is denoted that $t$ is the index of an arrow, and the traversals on arrows $arrow(t-2)$, $arrow(t-1)$, and $arrow(t)$ are performed in order. The calculations of steps on a diagonal arrow $arrow(t)$ do not have data dependency with each other. Each step calculation on $arrow(t)$ has data dependency with three steps on $arrow(t-1)$ and $arrow(t-2)$. For substitution operation, a step can be calculated after obtaining the step on $arrow(t-2)$. For insertion and deletion operations, two steps can be calculated depending on steps on $arrow(t-1)$. These calculations require the values of two steps for the substitution and insertion operations on the left column and one step for deletion operation on the same column.

For the explanation of the initialization in pseudocode, we have added the following explanation in lines 250~258 as:

> In the pseudocode, the procedure **Diagonal_Skipping** has two arguments: input sequence and pattern. Terms $i$, $j$, and $k$ denote the input sequence length, pattern length, and mismatch threshold $k$, respectively. Firstly, several elements in $(i+1) \times (j+1)$ two-dimensional array $D$ and pruning register $pruning\_reg$ are initialized. In this initialization, $D[0, ..., i][0]$ is initialized using only deletion operators. On the other hand, $D[0][1, ..., j]$ is initialized using only insertion operators. These steps can be simply calculated without considering $min()$ function in Eq (1). In the pruning register, the bit indicating the leftmost column (the 0-th column) is set as '1,' and other bits are set as '0.'

3. In the description of the distance D (beginning in line 75), the difference between x_i and X_i is unclear. Is X_i a string or a character? If it's a string, why is it indexed? If it's a character, why is it written with a capital letter (in contrast to lowercase letters in line 75).

***Answer***: Thanks for your sharp comments. During distance calculation, subsequence and subpattern are used, where we thought that the index was helpful for describing the subsequence of a sequence and Definition 1 defined the index. However, the previous draft missed the index for strings and caused the confusion between the length of strings and index of characters, so the expression has been revised in lines 75~78 as:

In [1], for strings $X_i = x_1, x_2, ..., x_{i-1}, x_i$ and $Y_j = y_1, y_2 ..., y_{j-1}, y_j$ where characters $x_a, y_b \in \mathbb{C}$ for $1 \leq a \leq i$ and $1 \leq b \leq j$, the distance between $X_i$ and $Y_j$, $D(X_i, Y_j)$ is the minimum number of edit operations to convert $X_i$ to $Y_i$. The distance $D(X_i, Y_j)$ should satisfy:

Additionally, in agreement with your concerns, we have inserted additional explanation in lines 98~110 as:

Black arrows 1, 2, and 3 in Fig 1 (a) means substitution, deletion, and insertion operators. which correspond to cost functions $substitution(x_\alpha, y_\beta)$, $deletion(x_\alpha)$, and $insertion(y_\beta)$ in Eq (1), respectively.

For example, for $\alpha = \beta = 3$, let's assume that $X_3$="bat" and $Y_3$="bad." In this case, by substituting $x_3$('$t$') with $y_3$('$d$') in $X_3$ in $substitution(x_3, y_3)$, the converted $X_3$ can be the same with $Y_3$, which adds the cost of $substitution$('$t$,' '$d$') to $D$("$ba$," "$ba$"). When $D$("$ba$," "$bad$") is given, character $x_3$('$t$') is removed from "bat." and the given $D$("$ba$," "$bad$") is required to convert "ba" to "bad." Therefore, the cost of $deletion$('$t$') is added to calculate $D$("$bat$," "$bad$"). When $D$("$bat$," "$ba$") is given, after attaching $y_3$='d' to "ba," $D$("$bat$," "$bad$") can be calculated, which means that the cost of $insertion$('$d$') should be added.

Therefore, the minimum edit distance for $X_3$="bat" and $Y_3$="bad" formulated as $D$("$bat$," "$bad$") is calculated on Eq (1) as:

$$D(\text{“}bat,\text{”}\ \text{“}bad\text{”}) = min \begin{cases} D(\text{“}ba,\text{”}\ \text{“}ba\text{”}) + substitution(\text{‘}t,\text{’}\ \text{‘}d\text{’}) \\ D(\text{“}ba,\text{”}\ \text{“}bad\text{”}) + deletion(\text{‘}t\text{’}) \\ D(\text{“}bat,\text{”}\ \text{“}ba\text{”}) + insertion(\text{‘}d\text{’}) \end{cases} \quad (2)$$

4. It appears the algorithm and the datasets used for the analysis are not publicly available.

***Answer***: We have uploaded our code used in the experiments in Github. (https://github.com/analog75/ED) . Besides, we have modified README.md to show the origin of English dictionary used in this evaluation. README.md in our Github is as:

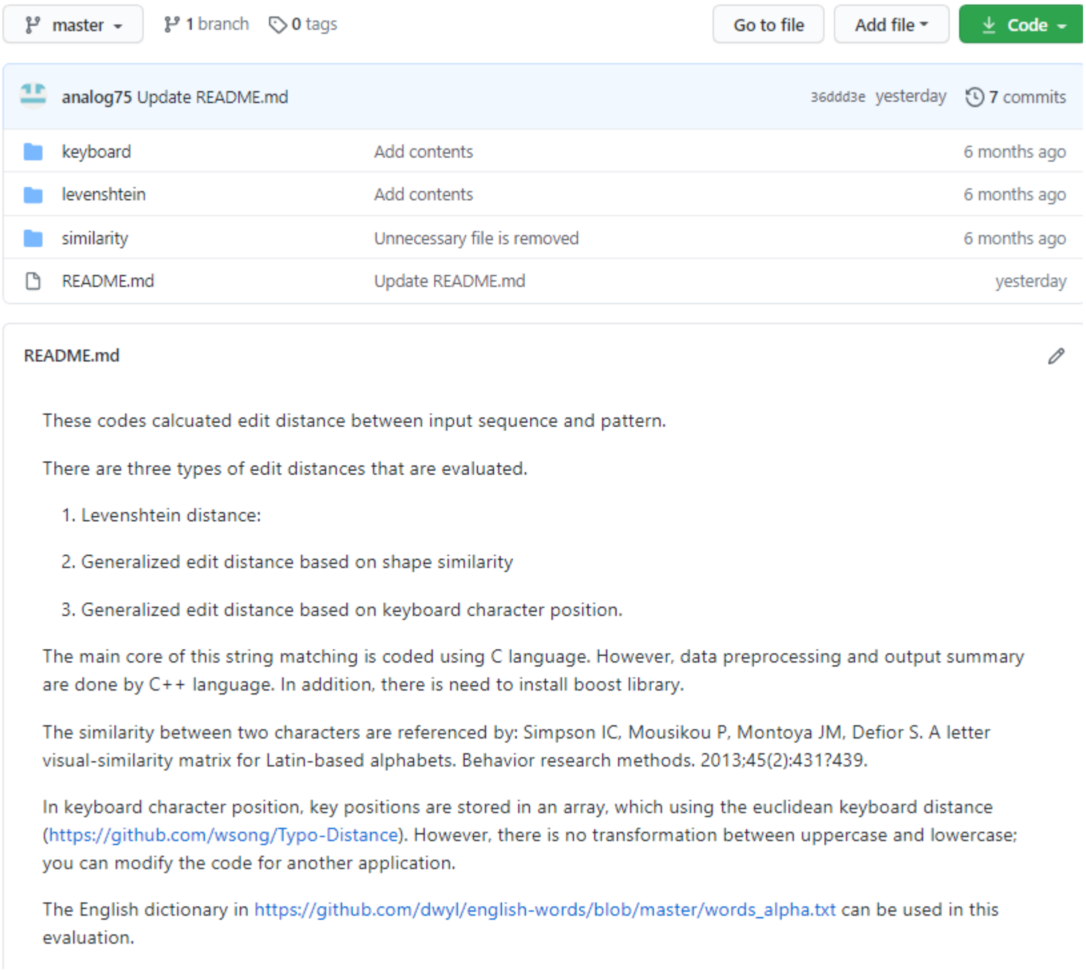

master | 1 branch | 0 tags | Go to file | Add file | Code

analog75 Update README.md | 36ddd3e yesterday | 7 commits

| | | |
|---|---|---|
| keyboard | Add contents | 6 months ago |
| levenshtein | Add contents | 6 months ago |
| similarity | Unnecessary file is removed | 6 months ago |
| README.md | Update README.md | yesterday |

README.md

These codes calcuated edit distance between input sequence and pattern.

There are three types of edit distances that are evaluated.

1. Levenshtein distance:
2. Generalized edit distance based on shape similarity
3. Generalized edit distance based on keyboard character position.

The main core of this string matching is coded using C language. However, data preprocessing and output summary are done by C++ language. In addition, there is need to install boost library.

The similarity between two characters are referenced by: Simpson IC, Mousikou P, Montoya JM, Defior S. A letter visual-similarity matrix for Latin-based alphabets. Behavior research methods. 2013;45(2):431?439.

In keyboard character position, key positions are stored in an array, which using the euclidean keyboard distance (https://github.com/wsong/Typo-Distance). However, there is no transformation between uppercase and lowercase; you can modify the code for another application.

The English dictionary in https://github.com/dwyl/english-words/blob/master/words_alpha.txt can be used in this evaluation.

5. The analysis part misses a statistical analysis. This can lead to a misinterpretation of the data: Shorter average running times do not necessarily imply a more efficient algorithm. Also, comparing running times per se can lead to systematic errors in the analysis. E.g. one of the algorithms might have been implemented more efficiently, the computer hardware might be more beneficial for one of the compared programs (e.g. due to cache structure, compiler optimization etc.).

***Answer***: : Thanks for your sharp comments. In agreement with your concerns, additional experiments and statistical analysis have been performed, where the explanation have been added in the revised version. With the additional experiments, we have shown how much step calculations can be skipped using the proposed method, which brought the critical relationship between the length of patterns and the execution time.

Firstly, evaluations have been performed by starting the measurement of the execution time after calling the procedure of string matching, where the overhead of transferring the parameters has been omitted to show the efficiency of the proposed algorithm more precisely. The revised experiments and their analysis are in lines 342~387 as :

Fig 6 shows the summary of average execution times by sweeping $k$ when using the Levenshtein distance metric. When the diagonal traversal did not adopt $k$-mismatches, the average execution time was longer than that of the dynamic programming using the vertical traversal because of the overhead from conditional statements and reordered memory accesses. In these experiments, the execution time increased with $k$. When $k > 4$, the execution time was over that of the vertical traversal, which means the proposed method did not have any benefits over the simple vertical traversal for large $k$. Besides, Fig 6 shows that the diagonal traversal without considering $k$-mismatches required additional overhead of conditional statements and reordered data accesses compared to the vertical traversal. Therefore, for the Levenshtein distance metric, when $k$ was small, we concluded that the proposed method can help reduce the execution time. Significantly, compared to the vertical and diagonal traversals, the execution times were decreased by 47.1% and 54.7% when $k = 1$.

For the generalized edit distance using similarity in shapes, Fig 7 illustrates the average execution times by sweeping $k$. Like the case using the Levenshtein distance metric, the execution time increased with $k$. When $k < 5$, the average execution times of the proposed diagonal skipping method were shorter than that of the vertical traversal, which means that many step calculations can be skipped for small $k$ in this generalized edit distance metric. The diagonal traversal only increased the average execution time by 11.5% over the vertical traversal. Notably, when $k = 1$, the execution times were reduced by 67.4% and 70.8% over the vertical and diagonal traversals. Compared to the evaluation using the Levenshtein distance metric, it was expected that the execution time can be further reduced because the overhead ratio of conditional statements and reordered memory accesses was smaller.

Fig 8 summarizes the average execution times based on the distance metric considering keyboard character positions. Like Figs 6 and 7, the execution times were evaluated by sweeping $k$. *Diagonal skipping(II)* adopted the skipping method using the pruning register and reduced unnecessary operator evaluations after accessing data-dependent previous steps over $k$. On the other hand, *diagonal skipping(I)* just used the skipping method using the pruning register. By avoiding unnecessary operator evaluations over $k$, the diagonal skipping(II) can further reduce the execution time when $k$ was small. As $k$ increased, the difference of the average execution time between diagonal skipping(I) and diagonal skipping(II) was reduced because the number of reduced operator evaluations using the diagonal skipping(II) diminished. When $k = 5$, the difference in the average execution times was negligible. When $k > 5$, the average execution time of diagonal skipping(II) was longer than that of diagonal skipping(I). Besides, when $k = 8$, the average execution time of diagonal skipping(II) was very close to those of the vertical and diagonal traversals. Like the Levenshtein distance and the generalized distance using similarity in shapes, many step calculations can be skipped for small $k$, and the number of skipped step calculations decreased with $k$. However, even when $k = 8$, the average execution time of the proposed method was shorter that those of the vertical and diagonal traversals. In agreement with Table 1, the computational costs of the operators used in this distance metric can be high compared to the conditional statements. Notably, compared to the vertical traversal, when $k = 1$, the diagonal skipping(I) and diagonal skipping(II) reduced the average execution time by 75.5% and 82.9%, respectively.

Secondly, we have performed experiments about the ratio of skipped edit distance calculation to know how much step calculations can be reduced using the proposed method in lines 388~397 as:

Fig 9 illustrates ratio of the skipped edit distance calculations. This experiment counted two types of skipped edit distance calculations when obtaining $D[\alpha][\beta]$. When the pruning bit of $(\beta - 1)$-th column was '1,' the insertion opertor was skipped, and only $D[\alpha - 1][\beta]$ and $D[\alpha - 1][\beta - 1]$ were accessed to calculate $D[\alpha][\beta]$. Secondly, if pruning bit of the $\beta$-th column was '1,' the next steps in a traversal were skipped because they were over $k$. When $k = 1$, 91.7$\sim$77.3% step calculations were skipped. As increasing $k$, the ratios decreased rapidly, where the decreasing ratios can be different depending on the adopted edit distance metric. When $k = 8$, only 11.4$\sim$3.7% step calculations can be skipped, where the overhead of conditional statements and reordered memory accesses increased the average execution time compared to the vertical and diagonal traversals.

Finally, the regression analysis of the proposed method has been performed to know the correlation between the execution time and other inputs such as lengths of input sequences and patterns, where the coefficient has shown that the lengths of patterns was critical in the execution time. The added explanation is in lines 398~416 as:

> The statistical analysis was performed to know the functional relationship between the execution time and input parameters. As shown in [36], the regression approach was adopted, and the input sequence and pattern lengths were used as input parameters. This evaluation was performed with $k = 2$ for all adopted edit distance metrics. In the regression analysis, the coefficients of determination ($R^2$) can be used to show how much the regression model was fit for the target data [37]. When using the Levenshtein distance metric, $R^2$ was just 0.214. On the other hand, the generalized edit distance metrics using similarity in shapes (denoted as Shape) and keyboard position (denoted as Keyboard) were 0.536 and 0.792, respectively. This results showed that except for the input sequence and pattern lengths, other overheads could affect the execution time significantly in the Levenshtein distance metric. Table 2 lists the results of the regression analysis for the adopted three edit distance metrics, where *Coef.*, *SE Coef.*, $T$, and $P$ denotes the coefficient, standard error coefficient, t-value, and p-value, respectively. Because the p-values were small, the input sequence and pattern lengths can be statistically significant. Large t-values in Table 2 shows that even though the input sequence and pattern lengths were the same, the execution time can be different severely depending on the values of the input sequence and pattern. Moreover, the coefficient for the pattern lengths was more significant than those of input sequences, which means that the pattern lengths were more critical in the execution time.

*Thanks for your advice, and your comments were very helpful to improve the quality of our paper.*

==============================================================

**Reviewer 2**:

Thank you for offering the opportunity to revise the paper PONE-D-20-11239. The paper has been revised with careful study in aspects of paper format, language, terminology, conveyed meaning, and grammar. In addition, the revised paper has addressed your concerns.

**Comments and Response:**

1. Some essential introductory notions are ambiguous and need to be better explained. For example, at line 75, according to the used notation, the input strings X and Y appear to have length i and j respectively, but afterwards, starting from line 76, i and j are used as indices. Even more confusion can be created by considering line 87, where the input sequence X and pattern Y are denoted with subscripts i and j, respectively.

***Answer***: Thanks for your sharp comments. In agreement with your concerns, the previous draft missed the index for strings and caused the confusion between the length of strings and index of characters, so several expressions have been revised.

Firstly, we have added the subscript for indicating the lengths of strings in lines 75~78 as:

In [1], for strings $X_i = x_1, x_2, ..., x_{i-1}, x_i$ and $Y_j = y_1, y_2 ..., y_{j-1}, y_j$ where characters $x_a, y_b \in \mathbb{C}$ for $1 \leq a \leq i$ and $1 \leq b \leq j$, the distance between $X_i$ and $Y_j$, $D(X_i, Y_j)$ is the minimum number of edit operations to convert $X_i$ to $Y_i$. The distance $D(X_i, Y_j)$ should satisfy:

Secondly, the indexes of the subsequence and subpatterns have been added for the clear meaning in lines 93~97 as:

**Definition 1** *For strings $X_\alpha = x_1, x_2, ..., x_{\alpha-1}, x_\alpha$, and $Y_\beta = y_1, y_2, ..., y_{\beta-1}, y_\beta$, when subscripts $\alpha \leq i$ and $\beta \leq j$, $X_\alpha$ and $Y_\beta$ are the input subsequence and subpattern of input sequence $X_i$ and pattern $Y_j$, respectively.*

For input subsequence $X_\alpha$ and subpattern $Y_\beta$, when the initial edit distance $D(X_0, Y_0)$ is 0, the minimum edit distance $D(X_\alpha, Y_\beta)$ is formulated as follows:

$$D(X_\alpha, Y_\beta) = min \begin{cases} D(X_{\alpha-1}, Y_{\beta-1}) + substitution(x_\alpha, y_\beta) \\ D(X_{\alpha-1}, Y_\beta) + deletion(x_\alpha) \\ D(X_\alpha, Y_{\beta-1}) + insertion(y_\beta). \end{cases} \quad (1)$$

Additionally, we have modified several expressions in this manuscript according to the definition and terms above.

2. English is generally very good, while the paper also has some typos and language issue which needs to be checked and corrected in the revision.

***Answer***: In agreement with your concerns, this manuscript has been proofread by a scientific editing service. Besides, grammatical errors and typos have been revised in this version.

3. Certain technical details should be expanded and clarified to ensure better understanding. For instance, in Equation 1 the functions of substitution, deletion and insertion should be precisely defined.

***Answer***: In agreement with your concerns, we have modified several terms and expression in Preliminary section in this revised version.

Notably, we have inserted additional explanation for understanding the indexes in this manuscript in lines 98~110 as:

Black arrows 1, 2, and 3 in Fig 1 (a) means substitution, deletion, and insertion operators. which correspond to cost functions $substitution(x_\alpha, y_\beta)$, $deletion(x_\alpha)$, and $insertion(y_\beta)$ in Eq (1), respectively.

For example, for $\alpha = \beta = 3$, let's assume that $X_3$="bat" and $Y_3$="bad." In this case, by substituting $x_3$('$t$') with $y_3$('$d$') in $X_3$ in $substitution(x_3, y_3)$, the converted $X_3$ can be the same with $Y_3$, which adds the cost of $substitution$('$t$,' '$d$') to $D$("$ba$," "$ba$"). When $D$("$ba$," "$bad$") is given, character $x_3$('$t$') is removed from "bat." and the given $D$("$ba$," "$bad$") is required to convert "ba" to "bad." Therefore, the cost of $deletion$('$t$') is added to calculate $D$("$bat$," "$bad$"). When $D$("$bat$," "$ba$") is given, after attaching $y_3$='d' to "ba," $D$("$bat$," "$bad$") can be calculated, which means that the cost of $insertion$('$d$') should be added.

Therefore, the minimum edit distance for $X_3$="bat" and $Y_3$="bad" formulated as $D$("$bat$," "$bad$") is calculated on Eq (1) as:

$$D(\text{“}bat,\text{”}\ \text{“}bad\text{”}) = min \begin{cases} D(\text{“}ba,\text{”}\ \text{“}ba\text{”}) + substitution(\text{‘}t,\text{’}\ \text{‘}d\text{’}) \\ D(\text{“}ba,\text{”}\ \text{“}bad\text{”}) + deletion(\text{‘}t\text{’}) \\ D(\text{“}bat,\text{”}\ \text{“}ba\text{”}) + insertion(\text{‘}d\text{’}) \end{cases} \quad (2)$$

*Thanks for your advice, and your comments were very helpful to improve the quality of our paper.*
