# Supplementary material for: A k-mismatch string matching for generalized edit distance using diagonal skipping method
Source: PLoS One. 2021 May 4;16(5):e0251047. doi: 10.1371/journal.pone.0251047 (PMC8096107; doi:10.1371/journal.pone.0251047)
Supplement: Attachment — Submitted filename: PLOSONE_2021_rebuttal(20210317).pdf [file pone.0251047.s002.pdf]

Response to the reviewers

Thank you for reviewing this paper PONE-D-20-11239R1 titles as ”A k-Mismatch String Matching for Generalized Edit Distance using Diagonal Skipping Method.” Considering reviewers’ comments, this paper has been revised with careful study in aspects of paper format, language, terminology, conveyed meaning, and grammar. Several typos and missing information have been corrected. In addition, the revised paper addressed all reviewers’ comments.

Reviewer 1

Point 1.1 — No concerns about the previous version.

Reviewer 2

Point 2.1 — A further accurate copy edit for language usage and grammar is mandatory. In fact a frequent mistake, is the usage of the verb in the third person singular when not required, for example at lines 98, 410, 412, but may not be the only ones. Other repetitive misspellings are the quoted comma (for example line 257: ‘1,’) and capital letter after comma (see line 16). There are also lexical inaccuracies as close statement repetitions (for instance 343-345 and 349-350) or unclear references (at 346-348 one does not promptly associate “these experiments” with “the diagonal skipping method”).

Reply: Thanks for your detail comments. We have corrected misspellings and grammatical errors from your concerns. Following the standard British usage, the comma has been outside the quote. Besides, this new version has been carefully rewritten to revise grammatical typos. Thanks again.

Point 2.2 — The distance (as defined in lines 76-77) is the minimum number of operations to convert the input string into the pattern, it follows that the operations are always applied on the input string and never on the pattern. Therefore in expression (1) should be correct $D(X_{\alpha-1}, Y_\beta) + insertion(y_\beta)$ and $D(X_\alpha, Y_{\beta-1}) + deletion(x_\alpha)$. This statement is also confirmed by the explanation of the example in Fig 1 (b), lines 112-118. Moreover the example in lines 104-109 needs a better adapted description.

Reply: Thanks for your sharp comments about the definition of the edit distance. We have reviewed the definitions from the on-line encyclopedia (e.g., Wikipedia) and several textbooks, where we have known that Eq (1) is commonly used for calculating the edit distance. In explanations, the insertion is used in the second string ($Y_{\beta-1}$). In order to keep the commonly used definition and agree with your concerns, the definition has been rewritten in the revised version (**lines 75-76**) as follows:

> The distance between X_i and Y_j denoted as $D(X_i, Y_j)$ is the minimum number of edit operations to make X_i and Y_i the same.

Besides, the additional explanation of the cost functions $substitution(x_\alpha, y_\beta)$, $deletion(x_\alpha)$, and $insertion(y_\beta)$ has been provided (**lines 99-102**) as:

> The function $substitution(x_\alpha, y_\beta)$ means the cost of substituting x_α of X_α into y_β of Y_β. The function $deletion(x_\alpha)$ provides the cost of deleting x_α from X. On the other hand, the function $insertion(y_\beta)$ means the cost of inserting y_β to the end of $Y_{\beta-1}$.

Point 2.3 — It is suggested to revisit the notations used to represent strings, substrings, the respective lengths and the indices to identify any character in the string/substring. There are misconceptions when the subscript refers to the length of a string/substring and when it refers to any character of the string/substring. For instance at line 267 the content suggests that $D[\alpha][1]$ wants to indicate the whole second column in the two-dimensional array D, however 'α' defines the length of the input substring. Moreover according to the employed notation $D[\alpha][\beta]$ (line 269) identifies only the rightmost bottom cell of D, but here it is intended to identify any cell of D.

Reply: Thanks for your detailed comments! As shown in *Preliminaries* section, X_α and Y_β mean the subsequence of input sequence X_i and subpattern of pattern Y_j for $1 \le \alpha \le i$ and $1 \le \beta \le j$. We think that this explaination will help understand the meaning of the terms α and β. Besides, this explanation will indicate that $D[\alpha][\beta]$ is not the edit distance of rightmost bottom, which has been added in this section (**lines 263-265**) as follows:

> As shown in *Preliminaries* section, X_α and Y_β mean the subsequence of input sequence X_i and subpattern of pattern Y_j for $1 \le \alpha \le i$ and $1 \le \beta \le j$.

In agreement with your concerns, in order to indicate the whole second column, we have rewritten several expressions (**lines 271-275**) as follows:

> Except for the calculation $D[s][1], 1 \le s \le i$, when the pruning bit of $(\beta - 1)$-th column is '1', the value stored in $D[\alpha][\beta - 1]$ is over k, so only $D[\alpha - 1][\beta]$ and $D[\alpha - 1][\beta - 1]$ are accessed to calculate $D[\alpha][\beta]$. When calculating $D[s][1], 1 \le s \le i$, all operators are considered because the pruning bit of the leftmost column is initialized as '1'.

Point 2.4 — Comparing the definition of 'traversal' specified in 120-121, its usage in the pseudocode and in lines 229-230, the difference between arrow and traversal is unsettled.

Reply: Thanks for your sharp comments. Whereas the steps in a diagonal traversal did not have data-dependency on each other, the steps in a vertical or horizontal traversal had data-dependency. Therefore, in agreement with your concerns, the definition of *traversal* should be redefined (**lines 121-123**) as:

> In a *traversal*, the steps included in the traversal are calculated in order. The traversal method determines the order of calculating steps.

The arrow just indicates the order of calculating steps in a traversal. In agreement with your concerns, $arrow$ in the explanation has been replaced by $traversal$ (**lines 234-236**) as follows:

> It is denoted that t is the index of a traversal, and the traversals indicated by arrows $traversal(t-2)$, $traversal(t-1)$, and $traversal(t)$ are performed in order.

Besides, the pseudocode of Algorithm 1 (**page 7**) has been revised as:

Algorithm 1 Diagonal Skipping

```
procedure DIAGONAL_SKIPPING(input sequence, pattern)
    Initialization(D[0, ..., i][0], D[0][1, ..., j], pruning_reg)
    for each traversal do
        for each step(α, β) ∈ traversal do
            if pruning_reg(β) == 1 then break;
            end if
            if pruning_reg(β − 1) == 0 then
                D[α][β] = cost_min((D[α − 1][β], D[α][β − 1], D[α − 1][β − 1]))
            else
                D[α][β] = cost_min((D[α − 1][β], D[α − 1][β − 1]))
            end if
            if D[α][β] > k and pruning_reg(β − 1) == 1 then
                pruning_reg(β) = 1;
            end if
        end for
    end for
    return D
end procedure
```

Thanks for your brilliant comments. We assure that your idea will be helpful for increasing the quality of this paper.
